# Investigation of the Trajectory of Muscle and Body Mass as a Prognostic Factor in Patients With Colorectal Cancer: Longitudinal Cohort Study

**DOI:** 10.2196/43409

**Published:** 2023-03-22

**Authors:** Dongjin Seo, Han Sang Kim, Joong Bae Ahn, Yu Rang Park

**Affiliations:** 1 Department of Medicine Yonsei University College of Medicine Seoul Republic of Korea; 2 Yonsei Cancer Center Division of Medical Oncology, Department of Internal Medicine Yonsei University College of Medicine Seoul Republic of Korea; 3 Brain Korea 21 FOUR Project Graduate School of Medical Science Yonsei University College of Medicine Seoul Republic of Korea; 4 Institute for Innovation in Digital Healthcare Severance Hospital Seoul Republic of Korea; 5 Department of Biomedical Systems Informatics Yonsei University College of Medicine Seoul Republic of Korea

**Keywords:** body mass index, BMI, colorectal cancer, deep neural network model, skeletal muscle, skeletal muscle volume index, SMVI

## Abstract

**Background:**

Skeletal muscle and BMI are essential prognostic factors for survival in colorectal cancer (CRC). However, there is a lack of understanding due to scarce studies on the continuous aspects of these variables.

**Objective:**

This study aimed to evaluate the prognostic impact of the initial status and trajectories of muscle and BMI on overall survival (OS) and assess whether these 4 profiles within 1 year can represent the profiles 6 years later.

**Methods:**

We analyzed 4056 newly diagnosed patients with CRC between 2010 to 2020. The volume of the muscle with 5-mm thickness at the third lumbar spine level was measured using a pretrained deep learning algorithm. The skeletal muscle volume index (SMVI) was defined as the muscle volume divided by the square of the height. The correlation between BMI status at the first, third, and sixth years of diagnosis was analyzed and assessed similarly for muscle profiles. Prognostic significances of baseline BMI and SMVI and their 1-year trajectories for OS were evaluated by restricted cubic spline analysis and survival analysis. Patients were categorized based on these 4 dimensions, and prognostic risks were predicted and demonstrated using heat maps.

**Results:**

Trajectories of SMVI were categorized as decreased (812/4056, 20%), steady (2014/4056, 49.7%), or increased (1230/4056, 30.3%). Similarly, BMI trajectories were categorized as decreased (792/4056, 19.5%), steady (2253/4056, 55.5%), or increased (1011/4056, 24.9%). BMI and SMVI values in the first year after diagnosis showed a statistically significant correlation with those in the third and sixth years (*P*<.001). Restricted cubic spline analysis showed a nonlinear relationship between baseline BMI and SMVI change ratio and OS; BMI, in particular, showed a U-shaped correlation. According to survival analysis, increased BMI (hazard ratio [HR] 0.83; *P*=.02), high baseline SMVI (HR 0.82; *P*=.04), and obesity stage 1 (HR 0.80; *P*=.02) showed a favorable impact, whereas decreased SMVI trajectory (HR 1.31; *P*=.001), decreased BMI (HR 1.23; *P*=.02), and initial underweight (HR 1.38; *P*=.02) or obesity stages 2-3 (HR 1.79; *P*=.01) were negative prognostic factors for OS. Considered simultaneously, BMI >30 kg/m^2^ with a low SMVI at the time of diagnosis resulted in the highest mortality risk. We observed improved survival in patients with increased muscle mass without BMI loss compared to those with steady muscle mass and BMI.

**Conclusions:**

Profiles within 1 year of both BMI and muscle were surrogate indicators for predicting the later profiles. Continuous trajectories of body and muscle mass are independent prognostic factors of patients with CRC. An automatic algorithm provides a unique opportunity to conduct longitudinal evaluations of body compositions. Further studies to understand the complicated natural courses of muscularity and adiposity are necessary for clinical application.

## Introduction

Colorectal cancer (CRC) is the third most common cancer, accounting for approximately 10% of newly diagnosed cancers and cancer-related mortality worldwide [1]. Epidemiologic studies indicate that increasing age and resource-rich countries are associated with CRC development [2]. Modifiable lifestyle factors such as smoking, alcohol intake, physical activity, and obesity possibly impact CRC development [3,4].

Given the rapid global increase in obesity in recent years, understanding the effects of obesity on cancer outcomes is important [5,6]. Previous studies suggest that increased BMI is associated with poor prognosis and resistance to anticancer treatment [7-9]. Patients with stage II or III disease with high BMI at the time of diagnosis had decreased overall survival (OS) and disease-free survival [10]. Moreover, studies have described a relationship between muscle and CRC prognosis, suggesting that low muscle mass (ie, sarcopenia) is a prognostic factor for poor survival, increased postoperative complications, and decreased treatment response [11,12]. Despite the well-established prognostic impact of baseline profiles of obesity and muscle mass, personalized risk assessments or intervention decisions regarding these body compositions are limited in clinical practice.

Previously, skeletal muscle was manually measured using computed tomography (CT) images at the level of the third lumbar spine vertebra (L3), which includes an intense localization procedure and inevitable operator errors in the manual identification of the L3 vertebrae [13,14]. Recently, the medical application of artificial intelligence (AI) using machine and deep learning algorithms has increased, enabling the resolution of medical problems with ease of use, robustness, and precision [15]. Moreover, studies have demonstrated that automated CT imaging evaluations using deep learning models are feasible for extracting complicated body composition parameters, and the prognostic effects of these measurements on mortality in CRC have been confirmed [16-18]. Although previous automated body profile evaluations have partially fulfilled the gap for clinical application, the evaluation of adiposity and muscularity is limited to one time point, and thus, not considered alternatively during various treatment or interventions.

To comprehend the natural course of adiposity and muscularity of patients with CRC, we continuously tracked skeletal muscle and BMI profiles over 1 year and evaluated their representativeness for longer prospective profiles. Moreover, we evaluated the prognostic effects of the trajectories of changes in skeletal muscle and BMI to identify their longitudinal effects on CRC prognosis and demonstrated predicted mortality risks for each body composition trajectory, specified by initial status and changes in BMI and muscularity. Our study highlights the continuous characteristics of muscle and BMI in OS and supports the clinical applicability of artificial intelligence–powered automated evaluation for CRC risk modification and management.

## Methods

### Study Population and Data Collection

A total of 4056 patients with newly diagnosed CRC were enrolled in the Yonsei Cancer Registry Database between January 1, 2010, and September 30, 2020. Patients with abdominal CT images and BMI information up to 1 year after diagnosis were eligible. CT images within 1 year of diagnosis were obtained from the medical database of Severance Hospital. BMI was measured as the patient’s weight in kilograms divided by the square of the patient’s height in meters (kg/m^2^). Skeletal muscle volume index (SMVI) outliers and SMVI change ratios were detected using median absolute deviation and were excluded. Demographic factors, such as age, sex, weight, height, variables related to diagnosis, progression, the treatment of CRC, and the date of death or follow-up loss, were collected.

### Automated CT-Derived Skeletal Muscle Mass Measurement

**Figure 1:**
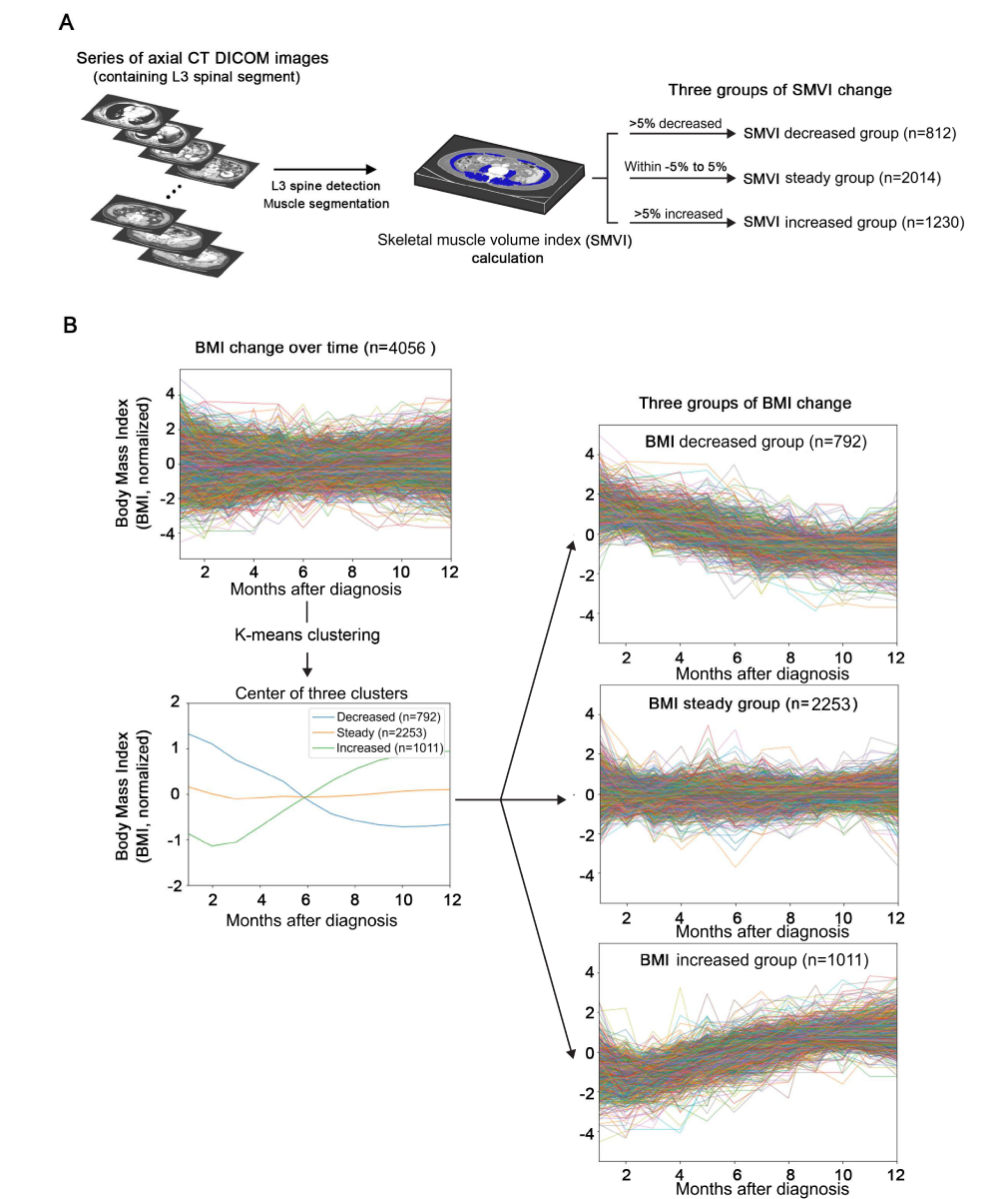
Study design. (A) The process of skeletal muscle segmentation and patient classification according to SMVI patterns. Axial CT image series were inputted (left). The blue area indicates the segmented muscle area and the volume of the muscle with 5-mm thickness was calculated (middle). Patients were classified into SMVI decreased, steady, and increased groups with the SMVI change ratio (right). (B) The process of patient classification according to 1-year BMI patterns. Lines indicate the 1-year BMI pattern of individual patients. The centers of 3 clusters by k-means clustering are presented. The 1-year BMI trajectories of the patients are demonstrated within each BMI pattern. CT: computed tomography; DICOM: Digital Imaging and Communications in Medicine; L3: third lumbar spine vertebra; SMVI: skeletal muscle volume index.

Using the UNet architecture–based automated skeletal muscle measurement algorithm proposed by Islam et al [19], skeletal muscle was analyzed with a series of axial CT images. The overall process of muscle assessment is summarized in Figure 1A. Automated CT-derived skeletal muscle mass measurement involved three steps: (1) axial CT images within 2.5 mm superior and inferior to L3 level were detected, (2) areas of the muscle were calculated, and (3) L3-level muscle volume was calculated. SMVI was defined as L3-level muscle volume divided by the square of the patient’s height in meters (cm^3^/m^2^).

### Patient Classification According to SMVI and BMI Patterns

Baseline SMVI was defined as the mean SMVI within 3 months after diagnosis. SMVI change ratios were calculated as the difference between the baseline and last SMVI values. SMVI patterns were divided into 3 groups according to the SMVI change ratio: below −5% as the decreased group, over 5% as the increased group, and between −5% to 5% as the steady group (Figure 1A). Patients’ 1-year BMI patterns were defined as the trajectory of the 12 monthly mean BMI values within 1 year after diagnosis. Missing monthly mean BMI was imputed assuming a linear change of monthly BMI. Using the k-means clustering method, patients were divided into 3 BMI pattern groups: decreased, steady, and increased (Figure 1B).

### Statistical Analysis

Patient characteristics were compared between BMI pattern groups. The normality of variables was determined using the quantile-quantile plot. Parametric and nonparametric continuous variables were compared using ANOVA and Kruskal-Wallis tests, respectively. Categorical variables were compared using the chi-square test. The effect sizes for ANOVA, Kruskal-Wallis test, and chi-square test were individually calculated using partial eta squared [20], eta squared [21], and Cramer V [22], respectively. The baseline BMI group was classified according to Asian cutoffs determined by the World Health Organization (WHO; BMI <18.5 kg/m^2^, underweight; 18.5-23 kg/m^2^, normal range; 23-25 kg/m^2^, preobese; 25-30 kg/m^2^, obese stage 1; and >30 kg/m^2^, obese stages 2-3) [23]. The SMVI was categorized into tertiles according to SMVI values (SMVI <18.68 cm^3^/m^2^ [lower third], low SMVI; 18.68-22.33 cm^3^/m^2^ [middle third], normal SMVI; and >22.33 cm^3^/m^2^ [upper third], high SMVI).

The correlation between the BMI of the first, third, and sixth years after diagnosis was evaluated with Pearson correlation, among patients with available BMI data for a sufficient period. Identical correlation analysis was conducted with SMVI among 300 random patients with available CT images for a sufficient period. The association of 6-year overall mortality with baseline and change ratio of both SMVI and BMI was analyzed using restricted cubic spline analysis. The nonlinearity was assessed by Wald statistics. Patients’ 6-year OS was analyzed for survival analysis, and survival curves were plotted using the Kaplan-Meier method and compared between SMVI pattern groups using the log-rank test. Cox proportional hazard regression was conducted to predict 6-year OS. Similar survival analyses were performed for each BMI pattern group. All statistical analyses were conducted using JupyterLab (version 1.2.6; Project Jupyter) [24], Python (version 3.6.8; Python Software Foundation) [25], and R (version 4.1.3; R Foundation for Statistical Computing) [26]. Two-sided *P* values of <.05 were considered statistically significant.

### Risk Assessment and Heat Map Generation

Three hazard ratio (HR) heat maps were designed with HRs of the survival analysis, with the *seaborn* package (v.0.11.2) [27]. One heat map demonstrated the HRs of 15 baseline conditions, defined by 5 baseline BMI and 3 baseline SMVI groups. The other heat map demonstrated the HRs of 9 body composition change patterns, defined by 3 BMI patterns and 3 SMVI pattern groups. Another heat map demonstrated the HRs of each baseline condition specified by the body composition change groups.

### Ethics Approval

The study design was approved by the institutional review board of Severance Hospital, Seoul, South Korea (IRB 4-2020-1304). The need for informed consent was waived by the ethics committee, as this study used routinely collected log data managed anonymously at all stages, including data cleaning and statistical analyses. The study protocol was performed following the guidelines of the International Conference on Harmonization of Good Clinical Practice, the Declaration of Helsinki, and relevant legislation for observational studies.

## Results

### Study Population

The median follow-up time was 45.5 (range 12.0-129.5) months. The median age was 61.0 (IQR 52.0-69.0) years, and 56.7% (2299/4056) of patients were men. At the time of diagnosis, the average baseline BMI was 23.2 (SD 3.0) kg/m^2^. Of the 4056 patients, 4.7% (n=192) were classified as underweight, 43.9% (n=1780) as normal weight, 25.1% (n=1019) as preobese, 23.9% (n=969) as obese stage 1, and 2.4% (n=96) as obese stages 2-3. The mean SMVI at diagnosis was 20.7 (SD 4.1) cm^3^/m^2^.

### Automated CT-Derived Skeletal Muscle Mass Measurement and Patient Classification Based on Longitudinal BMI and SMVI Changes

Patients were classified by SMVI patterns into 3 groups: decreased (812/4056, 20%), steady (2014/4056, 49.7%), and increased (1230/4056, 30.3%) groups. Using the k-means clustering method, clustering BMI patterns into 3 groups—decreased (792/4056, 19.5%), steady (2253/4056, 55.5%), and increased (1011/4056, 24.9%) groups—was the most optimized clustering option (Figure 1B and Multimedia Appendix 1) [28].

The baseline characteristics of each group according to BMI patterns are summarized in Table 1. The median age at diagnosis was 61.0 (IQR 52.0-69.0), 61.0 (IQR 53.0-69.0), and 60.0 (IQR 52.0-67.5) years in the decreased, steady, and increased BMI groups, respectively (Table 1). Baseline BMI was 24.4 (SD 3.1), 23.2 (SD 3.0), and 22.5 (SD 2.9) kg/m^2^ in the decreased, steady, and increased BMI groups, respectively. Baseline SMVI was 21.4 (SD 4.1), 20.8 (SD 4.1), and 20.0 (SD 3.8) cm^3^/m^2^ in the decreased, steady, and increased BMI groups, respectively.

**Table 1:** Patient characteristics according to 1-year BMI trajectory group: decreased, steady, and increased.

		1-year BMI trajectory pattern group			*P* value^a^		Effect size^b^	
		Decreased (n=792)	Steady (n=2253)	Increased (n=1011)				
Age (years), median (IQR)		61.0 (52.0-69.0)	61.0 (53.0-69.0)	60.0 (52.0-67.5)	.01		.002	
**Sex, n (%)**						.02		.044
	Male	474 (59.8)	1285 (57)	540 (53.4)				
	Female	318 (40.2)	968 (43)	471 (46.6)				
**Baseline, mean (SD)**								
	BMI (kg/m^2^)	24.4 (3.1)	23.2 (3.0)	22.5 (2.9)	<.001		.043	
	SMVI^c^ (cm^3^/m^2^)	21.4 (4.1)	20.8 (4.1)	20 (3.8)	<.001		.013	
**BMI group, n (%)**						<.001		.141
	Underweight	12 (1.5)	107 (4.7)	73 (7.2)				
	Normal	262 (33.1)	979 (43.5)	539 (53.3)				
	Preobese	206 (26)	604 (26.8)	209 (20.7)				
	Obesity stage 1	277 (35)	515 (22.9)	177 (17.5)				
	Obesity stages 2-3	35 (4.4)	48 (2.1)	13 (1.3)				
**1-year SMVI trajectory pattern group, n (%)**						<.001		.213
	Decreased	311 (39.3)	398 (17.7)	103 (10.2)				
	Steady	356 (44.9)	1219 (54.1)	439 (43.4)				
	Increased	125 (15.8)	636 (28.2)	469 (46.4)				
**Change ratio (%), mean (SD)**								
	BMI (kg/m^2^)	−9.1 (4.1)	0.4 (2.8)	11.0 (5.4)	<.001		.752	
	SMVI (cm^3^/m^2^)	−2.8 (9.1)	1.4 (7.8)	4.9 (8.7)	<.001		.088	
**Cancer stage, n (%)**						<.001		.116
	I	83 (10.5)	273 (12.1)	26 (2.6)				
	II	118 (14.9)	462 (20.5)	172 (17)				
	III	320 (40.4)	924 (41)	447 (44.2)				
	IV	271 (34.2)	594 (26.4)	366 (36.2)				
Recurrence or metastasis, n (%)		374 (47.2)	851 (37.8)	507 (50.1)	<.001		.113	
Death, n (%)		228 (28.8)	538 (23.9)	261 (25.8)	.02		.043	
Follow-up duration (years), mean (SD)^d^		3.7 (3.3)	4.0 (4.1)	3.8 (3.6)	<.001		.008	
**Treatment, n (%)**								
	Surgery	737 (93.1)	2071 (91.9)	902 (89.2)	.008		.049	
	CTx^e^	686 (86.6)	1822 (80.9)	961 (95.1)	<.001		.168	
	RTx^f^	377 (47.6)	613 (27.2)	256 (25.3)	<.001		.181	

^a^Categorical variables were compared using the chi-square test. Continuous variables were compared using ANOVA and Kruskal-Wallis tests.

^b^Cramer V effect size: 0.1=small, 0.3=medium, and 0.5=large (for 2 subvariable comparisons) and 0.07=small, 0.21=medium, and 0.35=large (for more than 3 subvariable comparisons). Partial eta-squared effect size: 0.01=small, 0.06=medium, and 0.14=large. Eta-squared effect size: 0.01=small, 0.06=medium, and 0.14=large.

^c^SMVI: skeletal muscle volume index.

^d^The follow-up duration of patients with a follow-up period longer than 6 years was considered as 6 years.

^e^CTx: chemotherapy.

^f^RTx: radiotherapy.

### Correlations Between First-Year and Subsequent Profiles of Obesity and Muscle

**Figure 2:**
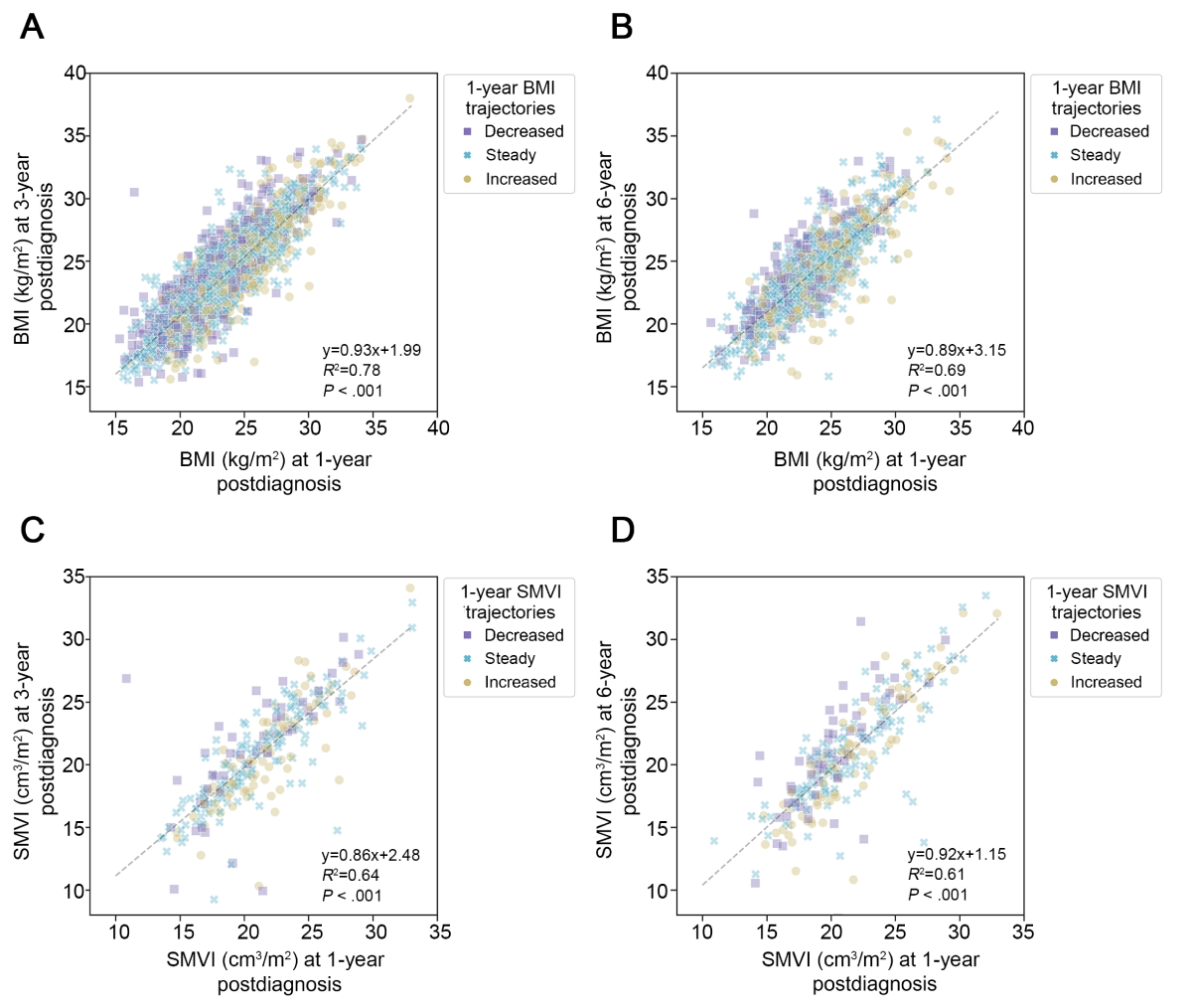
Correlation between BMI or SMVI profiles at the first year and 3 and 6 years after diagnosis. Scatter plots of patients’ BMI or SMVI profiles are presented, and results of trend line formula, R-square, and *P* value of Pearson correlation are described at the lower right in each graph. Magenta square, cyan cross, and yellow dot indicate decreased, steady, and increased groups, respectively, for the 1-year trajectory of BMI or SMVI. Black dashed lines represent the trend line of each scatter plots. SMVI: skeletal muscle volume index.

Among the 4056 patients, BMI data were available for 3217 patients 3 years after diagnosis and for 1318 patients 6 years after diagnosis. Among these patients, BMI in the first and third years showed a statistically significant positive correlation (*P*<.001), whereas the trajectory groups were evenly distributed (Figure 2A). BMI in the first year was also significantly correlated with that in the sixth year with a nonspecific distribution pattern of trajectory groups (*P*<.001; Figure 2B). Among the randomly selected 300 patients for correlation analysis between first- versus third-year SMVI, data from 278 patients were available for CT analysis, and the correlation was found to be positive (*P*<.001; Figure 2C). Among the randomly selected 300 patients for correlation analysis between first- versus sixth-year SMVI, 269 patients were analyzed, and the correlation was positive (*P*<.001; Figure 2D).

### Relationship of the Baseline and Change Ratio of Obesity and Muscle Mass With Mortality

**Figure 3:**
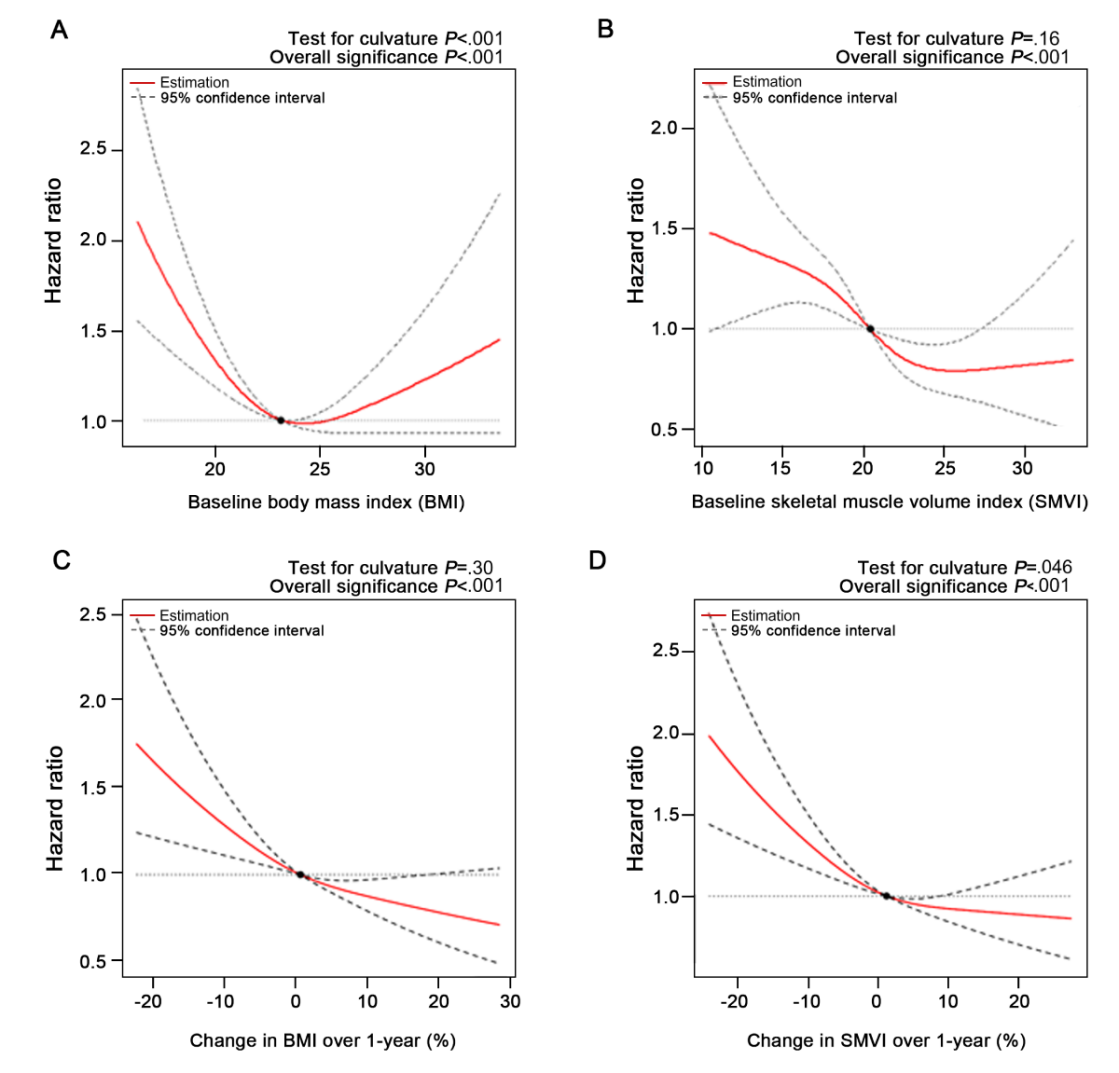
The relationship of the baseline and trajectories of obesity and muscle mass with mortality using restricted cubic spline analysis. Relationship between overall mortality and (A) baseline BMI, (B) baseline SMVI, (C) BMI change ratio, and (D) SMVI change ratio. *P* values of nonlinearity and significance of the relationship are described on top of each graph. Red lines represent restricted cubic spline curves, and black dashed lines represent 95% CIs. The reference is the median of each variable. Adjusted variables were age at diagnosis (above or below 65 years); sex; stage; primary cancer location (colon or rectum); histology (adenocarcinoma or others); recurrence or metastasis; the administration of surgery, chemotherapy, or radiotherapy; baseline BMI (underweight, normal, preobese, obesity stage 1, or obesity stages 2-3); baseline SMVI (low, normal, or high); and patterns of BMI and SMVI (decreased, steady, or increased). Baseline BMI, baseline SMVI, BMI pattern, and SMVI pattern were excluded from an adjustment in the analysis against baseline BMI, baseline SMVI, BMI change ratio, and SMVI change ratio, respectively. SMVI: skeletal muscle volume index.

According to restricted cubic spline analysis, baseline BMI and mortality risk exhibited a U-shaped relationship (*P*<.001; Figure 3A). The lowest mortality risk was observed among patients with a normal BMI. Baseline SMVI exhibited an inverse relationship with the risk of death, with an L-shaped pattern, whereby patients with the lowest muscle mass had the highest risk (*P*<.001; Figure 3B).

Change ratios of BMI demonstrated an inverse correlation with overall mortality (*P*<.001; Figure 3C), with the lowest risk observed for an increased BMI of >20%. Patients with BMI loss >20% had a 65% increased risk of death. Similarly, an increase in SMVI over time was associated with improved OS (*P*<.001; Figure 3D). A decrease in SMVI by 20% increased the mortality risk by 78%, suggesting that the restoration of body mass and muscle is critical for improving OS.

### BMI and SMVI as Prognostic Factors for Patient Outcomes in Multivariate Analysis

**Figure 4:**
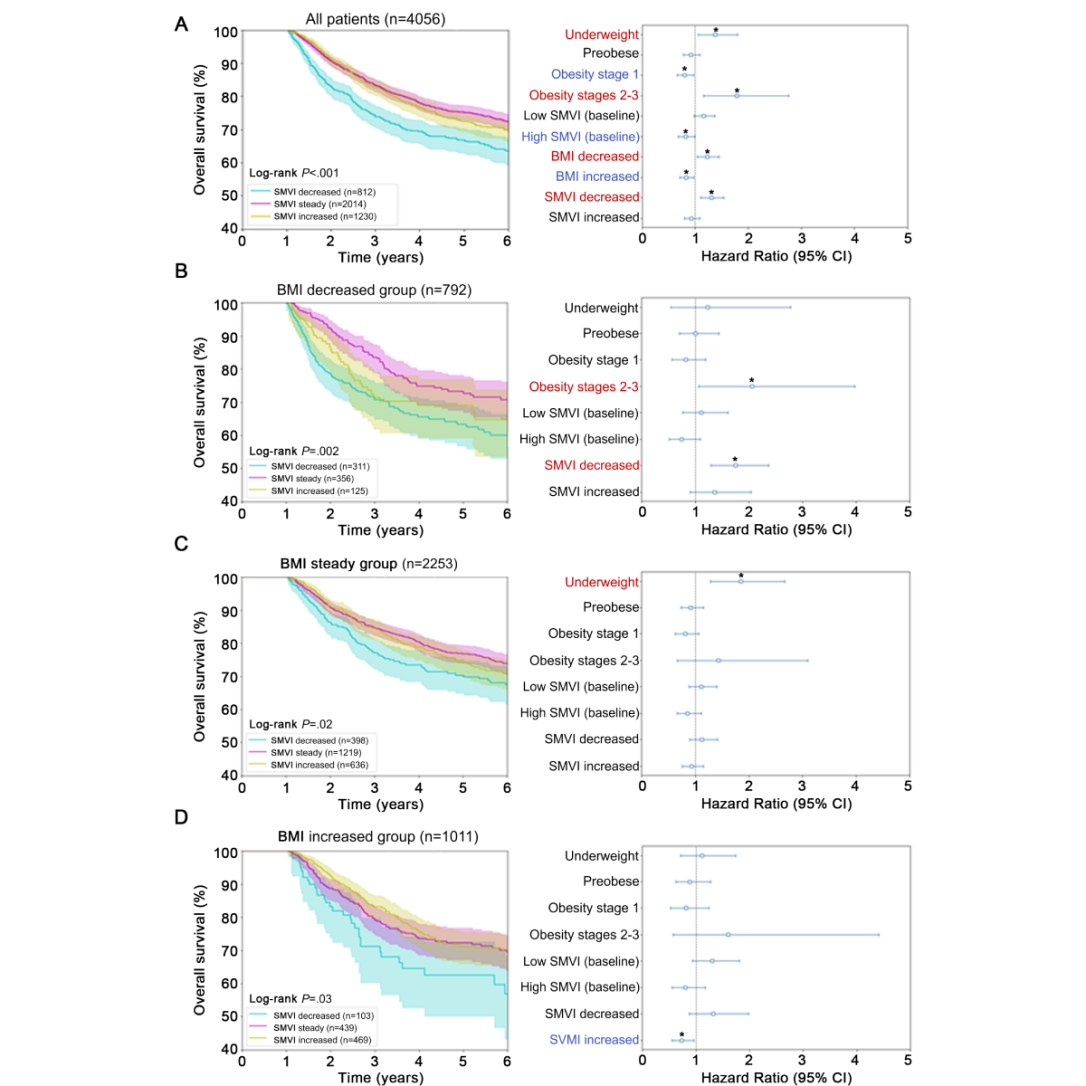
Kaplan-Meier curve and Cox proportional hazard regression analysis. Results for (A) total patients, (B) decreased BMI group, (C) steady BMI group, and (D) increased BMI group are presented. Kaplan-Meier curve results are shown on the left. Cyan, magenta, and yellow lines indicate decreased, steady, and increased SMVI groups, respectively. The hazard ratio and 95% CIs are shown on the right. Red and blue marked variables are statistically significant (*P*<.05) with hazard ratios of >1 and <1, respectively. Variables denoted with stars showed statistically significant *P* values (<.05). Adjusted variables were age at diagnosis (above or below 65 years); sex; stage; primary cancer location (colon or rectum); histology (adenocarcinoma or others); recurrence or metastasis; the administration of surgery, chemotherapy, or radiotherapy; baseline BMI (underweight, normal, preobese, obesity stage 1, or obesity stages 2-3); baseline SMVI (low, normal, or high); and patterns of BMI and SMVI (decreased, steady, or increased). SMVI: skeletal muscle volume index.

The trajectory of the decreased SMVI group was associated with shorter OS (6-year OS rate: 63.4% in decreased SMVI vs 72.4% in steady SMVI vs 69.6% in increased SMVI; *P*<.001). Multivariate analysis indicated that obesity stage 1 (HR 0.80, 95% CI 0.66-0.97; *P*=.02), high baseline SMVI (HR 0.82, 95% CI 0.68-0.99; *P*=.04), and increased BMI (HR 0.83, 95% CI 0.71-0.97; *P*=.02) were favorable prognostic factors for OS. Negative prognostic factors included being underweight (HR 1.38, 95% CI 1.06-1.80; *P*=.02), obesity stages 2-3 (HR 1.79, 95% CI 1.16-2.76; *P*=.008), decreased BMI (HR 1.23, 95% CI 1.04-1.45; *P*=.02), and decreased SMVI (HR 1.31, 95% CI 1.11-1.54; *P*=.001), consistent with the restricted cubic spline analysis (Figure 4A and Multimedia Appendix 2).

Based on the trajectories of BMI changes affecting OS, we performed a subgroup analysis according to 3 BMI trajectories (Figure 4B-D). The trajectory of SMVI changes impacted OS in the 3 BMI change groups (log-rank test: decreased BMI, *P*=.002; steady BMI, *P*=.02; and increased BMI, *P*=.03; Figure 4B-D). Of note, in patients with a decrease in BMI, the analysis revealed that obesity stages 2-3 at diagnosis (HR 2.06, 95% CI 1.07-3.97; *P*=.03) and decreased SMVI (HR 1.75, 95% CI 1.30-2.37; *P*<.001) had a negative prognostic impact on OS, suggesting that increased body fat at diagnosis or muscle wasting was associated with unfavorable outcomes in CRC (Figure 4B and Multimedia Appendix 3). In the steady BMI group, being underweight at the time of CRC diagnosis (HR 1.85, 95% CI 1.29-2.67; *P*=.001) had an adverse prognostic impact on OS (Figure 4C and Multimedia Appendix 4). In the increased BMI group, an increase in SMVI was associated with better OS (HR 0.73, 95% CI 0.55-0.97; *P*=.03), suggesting that increased body mass, predominantly due to skeletal muscle mass, positively impacted cancer survival (Figure 4D and Multimedia Appendix 5).

### HR Heatmap Representing Associations Between BMI, SMVI, and OS

**Figure 5:**
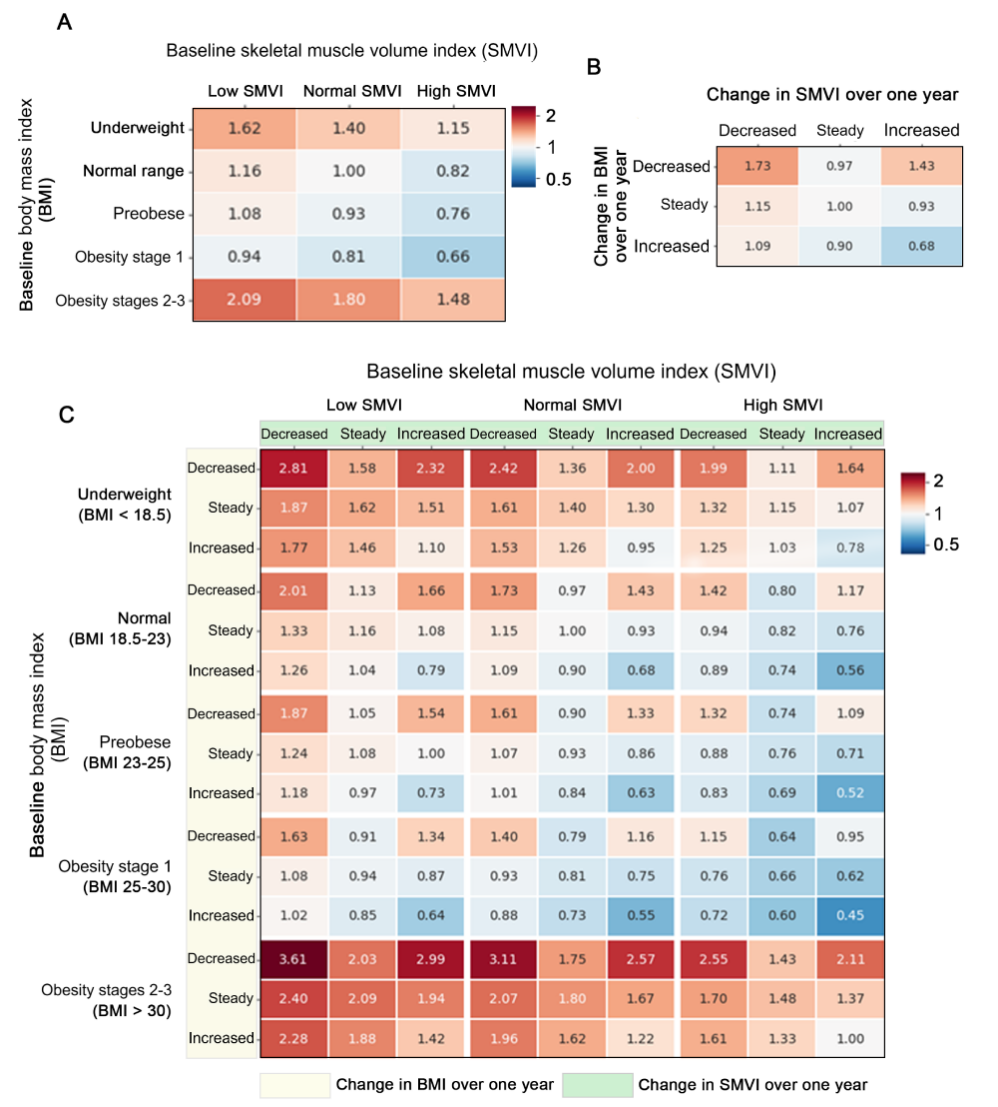
Predicted mortality risk heat map representing associations between BMI, skeletal muscle volume index (SMVI), and overall survival (OS). Patient groups of each heat map were classified by (A) baseline status classified by 5 baseline BMI and 3 baseline SMVI groups, (B) pattern groups classified by 3 BMI and 3 SMVI pattern groups, and (C) all 4 dimensions: baseline BMI and SMVI profiles and trajectories of BMI and SMVI. The predicted mortality risk of each specific patient group is described in each corresponding square, and colors represent the natural logarithm of the predicted mortality risk.

In consideration of baseline BMI and SMVI at the time of diagnosis, the highest mortality risk was observed in patients with high body mass (BMI >30 kg/m^2^) and low SMVI (HR 2.09; Figure 5A), although high muscle mass at the time of CRC diagnosis had a positive impact on OS (HR range 0.66-0.82; BMI 18.5-30 kg/m^2^). Correlation analysis between the trajectories of BMI and SMVI revealed that increased body mass and muscle mass were associated with the lowest mortality risk (HR 0.68; *P*=.001; Figure 5B), whereas decreased body mass and muscle wasting were associated with the highest mortality risk (HR 1.73; *P*<.001; Figure 5B and Multimedia Appendices 6 and 7).

Finally, we generated an HR heat map depicting the baseline and trajectories of both BMI and SMVI (Figure 5C). High baseline BMI (>30 kg/m^2^) was associated with increased mortality risk regardless of baseline muscle mass or muscle changes (Figure 5C). High baseline SMVI was associated with improved survival in patients with a BMI range from normal to obesity stage 1. There was a trend toward improved OS in patients with increased muscle without BMI loss.

## Discussion

### Principal Findings

This study evaluated the characteristics of baseline BMI and SMVI and their 1-year trajectories along with the prognostic impacts of these 4 parameters on OS in patients with CRC. The BMI and SMVI profiles of the first year after diagnosis were highly correlated with the third- and sixth-year profiles, implying that these are surrogate indicators for representing subsequent body composition profiles. Moreover, our survival analysis results indicated that high muscularity positively impacted OS, whereas both depletion and excess adiposity adversely impacted survival. Alterations in adiposity and muscle mass significantly affected 6-year overall mortality. Reciprocal compensation of these 2 factors indicated that changes in BMI had a superior prognostic impact than changes in muscle.

### Comparison With Prior Work

According to our restricted cubic spline analysis, baseline SMVI did not exhibit a nonlinear relationship with mortality, whereas baseline BMI demonstrated a U-shaped relationship with mortality. In addition, high muscle mass at baseline exhibited a protective effect against survival, and extreme BMI (underweight or severe obesity) predicted lower survival. These findings suggest that muscularity at the time of diagnosis may have protective effects, whereas both adiposity depletion and excess may have disadvantages for survival. The results of the survival analysis of our study also support the independent prognostic effects of body composition profiles at the time of diagnosis. The mechanisms underscoring the impact of body composition on CRC mortality at the time of diagnosis have yet to be elucidated because completely eliminating methodological biases is challenging [8,9]. A widely accepted explanation is that an adequate amount of adiposity acts as a metabolic reservoir and allows patients to tolerate cachectic situations during cancer progression and treatment [29]. However, excess body fat is correlated with higher mortality via various pathways. Dysregulated adipose tissue can increase inflammatory adipokines, leading to systemic inflammation and a tumor-friendly microenvironment [30], thereby exacerbating catabolic pathways [31]. Patients with low muscularity and high adiposity were associated with higher inflammatory-related serum proteins and cytokines [17]. In addition, the specification of adipose tissue into subcutaneous, visceral, and intra- or intermuscular adipose tissue revealed distinct relationships with CRC mortality: subcutaneous fat exhibits a U-shaped association, whereas visceral and intermuscular fats exhibit a positive relationship, implying context-dependent roles of fat [9].

In this study, an increase in muscle mass or BMI demonstrated higher OS, highlighting the beneficial effects of increasing muscle and body mass. Survival analysis within subgroups of each BMI trajectory group showed a combined prognostic influence of muscularity and adiposity. HR results of the survival analysis conducted within steady and increased BMI groups described that survival improved in the order of increased, steady, and decreased SMVI. However, within the decreased BMI group, an increase in SMVI was associated with poor survival compared with steady SMVI, which contradicts general findings. Although several findings were statistically insignificant, this implies that the protective role of increased muscle differs regarding the characteristics of BMI changes, since the prognostic effects of changes in muscle and adiposity may reciprocally compensate. In addition, according to the survival analysis considering all 9 patterns of BMI and SMVI change, patients in the increased SMVI with decreased BMI group had statistically significant increased mortality risk. The superior decrease of adiposity that leads to decreased BMI despite the improvement of muscularity may override the protective effects of increased muscle mass.

Although BMI is known to have limitations in the direct representation of adiposity [9], BMI profiles within 1 year could be an efficient parameter for predicting the prognosis of patients with CRC in most primary care settings. First, continuously repeated measurements of BMI can be performed, which enables longitudinal evaluation with ease. Second, a 1-year BMI profile highly correlates with future BMI profiles. Third, the trajectory of BMI is a critical independent prognostic factor, where it is a parameter containing longitudinal information of multiple compositions. Adiposity-related [17] and muscle-related indexes such as SMVI, muscle-to-bone ratio, or skeletal muscle radiodensity [16,18] are also essential prognostic factors, yet frequent assessments are limited since these parameters are products of occasional CT imaging studies that depend on cancer treatment progress, patient’s medical condition, health insurance, etc. Thus, synergizing CT-driven information and BMI data will promote the future clinical application.

Previous studies support the association between physical activity and cancer prognosis. A prospective cohort study revealed that sedentary behavior is associated with cancer mortality risk and replacing sedentary time with physical activities may improve OS [32]. According to a meta-analysis of nonrandomized trials, higher-intensity physical activity was significantly associated with lower mortality risk [33]. In addition, resistance training in patients with cancer resulted in improvement in muscle strength, body composition, and benefits for cancer survival [34,35]. Exercise is safe and feasible for patients with CRC and results in improvements in various health-related outcomes [36]. Collectively, appropriate interventions through physical activities could improve the outcomes of patients with CRC by improving body composition profiles. Studies evaluating the role of an exercise intervention to improve OS are rare; however, a phase-2 randomized clinical trial proved that high-intensity interval training delayed prostate cancer progression [37]. Thus, further studies evaluating whether exercise intervention improves long-term outcomes should be conducted.

### Strengths

Our study has the following strengths. We analyzed a large size of patients and assessed various natural courses of patients with CRC who did not receive additional interventions besides clinical practices. To comprehend the role of adiposity and muscularity in CRC progression, understanding its alternating nature is necessary for evaluating time-dependent prognostic impacts. Therefore, longitudinal patterns of BMI and muscle were analyzed simultaneously. Given that adiposity and muscle commonly change during cancer treatments [9], we longitudinally analyzed BMI and muscle for up to 1 year, and our study shows that the 1-year profile of 2 variables can aptly represent the course over 6 years. As these are proven to be independent prognostic factors, analyzing the 1-year course of adiposity and muscularity will be an efficient and effective method to determine the mortality risk. As a part of continuous body composition analysis, the deep learning–based assessment was implemented to minimize manual processes and enable rapid and accurate analysis of a large number of CT images. The application of automated analysis processes in clinical practice will highly promote the improvement of personalized care and risk management.

### Limitations

Our study has several limitations. We evaluated the 6-year OS as a survival outcome using 1-year trajectories of BMI and muscle mass. However, according to the correlation analysis, trajectory analysis up to 1 year was sufficient for representing the courses beyond a year. Since physical examinations and imaging studies are frequent during the first year as a part of the diagnosis or treatment process, we assumed that 1 year was sufficient for classifying body composition profiles. Prolonged longitudinal trajectorial analysis may provide additional insight into the longer-term effects of adiposity or muscle in cancer survival, thereby facilitating personalized risk assessment and management.

### Conclusions

In conclusion, our findings demonstrated the natural courses of BMI and muscle and highlight the 1-year trajectory as a surrogate indicator for representing further progress, wherein deep learning–based automated muscle assessment enabled longitudinal analysis of muscle. Thus, this study can be ground evidence for further research regarding prognostic improvements of patients with cancer by interventions that improve body composition profiles. In addition, our results provided crucial insights into the prognostic roles of adiposity and muscle mass in CRC survival. Body composition profiles at the time of diagnosis and alterations of these parameters are independent prognostic factors of CRC survival. The combination of BMI, which is frequently gathered but crude, with muscle-related parameter, which is automatically analyzed but occasionally obtained, could be a precise risk assessment tool. For further clinical application, supporting studies evaluating the natural course of patients with CRC will be necessary and more personalized prognosis prediction methods are required. To fully understand the role of adiposity and muscularity in cancer progression, impacts on other clinical outcomes should also be studied.

## Appendix

Multimedia Appendix 1 The inertia result of the k-means clustering of patients’ 1-year trajectory according to cluster numbers from 1 to 10. The most efficient clustering result was 3 clusters using the elbow method, and its inertia is denoted by the red circle.

Multimedia Appendix 2 Cox proportional hazard regression result within total patients. Adjusted variables were age at diagnosis (above or below 65 years); sex; stage; primary cancer location (colon or rectum); histology (adenocarcinoma or others); recurrence or metastasis; the administration of surgery, chemotherapy, or radiotherapy; baseline BMI (underweight, normal, preobese, obesity stage 1, or obesity stages 2-3); baseline SMVI (low, normal, or high); and patterns of BMI and SMVI (decreased, steady, or increased). SMVI: skeletal muscle volume index.

Multimedia Appendix 3 Cox proportional hazard regression result within the decreased BMI group. Adjusted variables were age at diagnosis (above or below 65 years); sex; stage; primary cancer location (colon or rectum); histology (adenocarcinoma or others); recurrence or metastasis; the administration of surgery, chemotherapy, or radiotherapy; baseline BMI (underweight, normal, preobese, obesity stage 1, or obesity stages 2-3); baseline SMVI (low, normal, or high); and patterns of SMVI (decreased, steady, or increased). SMVI: skeletal muscle volume index.

Multimedia Appendix 4 Cox proportional hazard regression result within the steady BMI group. Adjusted variables were age at diagnosis (above or below 65 years); sex; stage; primary cancer location (colon or rectum); histology (adenocarcinoma or others); recurrence or metastasis; the administration of surgery, chemotherapy, or radiotherapy; baseline BMI (underweight, normal, preobese, obesity stage 1, or obesity stages 2-3); baseline SMVI (low, normal, or high); and patterns of SMVI (decreased, steady, or increased). SMVI: skeletal muscle volume index.

Multimedia Appendix 5 Cox proportional hazard regression result within the increased BMI group. Adjusted variables were age at diagnosis (above or below 65 years); sex; stage; primary cancer location (colon or rectum); histology (adenocarcinoma or others); recurrence or metastasis; the administration of surgery, chemotherapy, or radiotherapy; baseline BMI (underweight, normal, preobese, obesity stage 1, or obesity stages 2-3); baseline SMVI (low, normal, or high); and patterns of SMVI (decreased, steady, or increased). SMVI: skeletal muscle volume index.

Multimedia Appendix 6 Cox proportional hazard regression result for hazard ratio heat map. Adjusted variables were age at diagnosis (above or below 65 years); sex; stage; primary cancer location (colon or rectum); histology (adenocarcinoma or others); recurrence or metastasis; the administration of surgery, chemotherapy, or radiotherapy; baseline BMI (underweight, normal, preobese, obesity stage 1, or obesity stages 2-3); baseline SMVI (low, normal, or high); and 9 patient groups divided by three 1-year BMI trajectories (decreased, steady, or increased) and three 1-year SMVI trajectories (decreased, steady, or increased). SMVI: skeletal muscle volume index.

Multimedia Appendix 7 Survival analysis results against total patients for hazard ratio heat map. (A) Kaplan-Meier curve and survival curve comparison by log-rank test. Patient groups were classified with 1-year trajectories of BMI and SMVI and are represented in different colors. (B) Cox proportional hazard regression result for hazard ratio heat map. Adjusted variables were age at diagnosis (above or below 65 years); sex; stage; primary cancer location (colon or rectum); histology (adenocarcinoma or others); recurrence or metastasis; the administration of surgery, chemotherapy, or radiotherapy; baseline BMI (underweight, normal, preobese, obesity stage 1, or obesity stages 2-3); baseline SMVI (low, normal, or high); and 9 patient groups divided by three 1-year BMI trajectories (decreased, steady, or increased) and three 1-year SMVI trajectories (decreased, steady, or increased). SMVI: skeletal muscle volume index.

## Abbreviations

CRC: colorectal cancer
CT: computed tomography
HR: hazard ratio
L3: third lumbar spine vertebra
OS: overall survival
SMVI: skeletal muscle volume index
WHO: World Health Organization

## Data Availability

Raw data for this study were generated at Severance Hospital. Derived data supporting the findings of this study are available from the corresponding author upon request.

## References

[1] Sung H, Ferlay J, Siegel RL, Laversanne M, Soerjomataram I, Jemal A, Bray F (2021). Global Cancer Statistics 2020: GLOBOCAN Estimates of Incidence and Mortality Worldwide for 36 Cancers in 185 Countries. CA Cancer J Clin.

[2] Dekker E, Tanis PJ, Vleugels JLA, Kasi PM, Wallace MB (2019). Colorectal cancer. Lancet.

[3] Khil H, Kim SM, Hong S, Gil HM, Cheon E, Lee DH, Kim YA, Keum N (2021). Time trends of colorectal cancer incidence and associated lifestyle factors in South Korea. Sci Rep.

[4] Kuipers EJ, Grady WM, Lieberman D, Seufferlein T, Sung JJ, Boelens PG, van de Velde CJH, Watanabe T (2015). Colorectal cancer. Nat Rev Dis Primers.

[5] Moghaddam AA, Woodward M, Huxley R (2007). Obesity and risk of colorectal cancer: a meta-analysis of 31 studies with 70,000 events. Cancer Epidemiol Biomarkers Prev.

[6] Bhaskaran K, Douglas I, Forbes H, dos-Santos-Silva I, Leon DA, Smeeth L (2014). Body-mass index and risk of 22 specific cancers: a population-based cohort study of 5.24 million UK adults. Lancet.

[7] Caan BJ, Meyerhardt JA, Kroenke CH, Alexeeff S, Xiao J, Weltzien E, Feliciano EC, Castillo AL, Quesenberry CP, Kwan ML, Prado CM (2017). Explaining the obesity paradox: the association between body composition and colorectal cancer survival (C-SCANS study). Cancer Epidemiol Biomarkers Prev.

[8] Cespedes Feliciano EM, Kroenke CH, Caan BJ (2018). The obesity paradox in cancer: how important is muscle?. Annu Rev Nutr.

[9] Caan BJ, Cespedes Feliciano EM, Kroenke CH (2018). The importance of body composition in explaining the overweight paradox in cancer-counterpoint. Cancer Res.

[10] Renfro LA, Loupakis F, Adams RA, Seymour MT, Heinemann V, Schmoll H, Douillard J, Hurwitz H, Fuchs CS, Diaz-Rubio E, Porschen R, Tournigand C, Chibaudel B, Falcone A, Tebbutt NC, Punt CJA, Hecht JR, Bokemeyer C, Van Cutsem E, Goldberg RM, Saltz LB, de Gramont A, Sargent DJ, Lenz H (2016). Body mass index is prognostic in metastatic colorectal cancer: pooled analysis of patients from first-line clinical trials in the ARCAD database. J Clin Oncol.

[11] Baracos VE, Arribas L (2018). Sarcopenic obesity: hidden muscle wasting and its impact for survival and complications of cancer therapy. Ann Oncol.

[12] Abbass T, Dolan R, McSorley ST, Horgan PG, McMillan DC (2020). Skeletal muscle index (SMI) status and survival in patients undergoing surgery for colorectal cancer (CRC): A longitudinal study. J Clin Oncol.

[13] Derstine BA, Holcombe SA, Ross BE, Wang NC, Su GL, Wang SC (2021). Optimal body size adjustment of L3 CT skeletal muscle area for sarcopenia assessment. Sci Rep.

[14] Vangelov B, Bauer J, Kotevski D, Smee RI (2022). The use of alternate vertebral levels to L3 in computed tomography scans for skeletal muscle mass evaluation and sarcopenia assessment in patients with cancer: a systematic review. Br J Nutr.

[15] Rajkomar A, Dean J, Kohane I (2019). Machine learning in medicine. N Engl J Med.

[16] Keyl J, Hosch R, Berger A, Ester O, Greiner T, Bogner S, Treckmann J, Ting S, Schumacher B, Albers D, Markus P, Wiesweg M, Forsting M, Nensa F, Schuler M, Kasper S, Kleesiek J (2023). Deep learning-based assessment of body composition and liver tumour burden for survival modelling in advanced colorectal cancer. J Cachexia Sarcopenia Muscle.

[17] Fleming CA, O'Connell EP, Kavanagh RG, O'Leary DP, Twomey M, Corrigan MA, Wang JH, Maher MM, O'Connor OJ, Redmond HP (2021). Body composition, inflammation, and 5-year outcomes in colon cancer. JAMA Netw Open.

[18] Xiao J, Caan BJ, Cespedes Feliciano EM, Meyerhardt JA, Peng PD, Baracos VE, Lee VS, Ely S, Gologorsky RC, Weltzien E, Kroenke CH, Kwan ML, Alexeeff SE, Castillo AL, Prado CM (2020). Association of low muscle mass and low muscle radiodensity with morbidity and mortality for colon cancer surgery. JAMA Surg.

[19] Islam S, Kanavati F, Arain Z, Da Costa OF, Crum W, Aboagye EO, Rockall AG (2022). Fully automated deep-learning section-based muscle segmentation from CT images for sarcopenia assessment. Clin Radiol.

[20] Lakens D (2013). Calculating and reporting effect sizes to facilitate cumulative science: a practical primer for t-tests and ANOVAs. Front Psychol.

[21] Tomczak M, Tomczak E (2014). The need to report effect size estimates revisited: an overview of some recommended measures of effect size. Trends Sport Sci.

[22] Kim HY (2017). Statistical notes for clinical researchers: chi-squared test and Fisher's exact test. Restor Dent Endod.

[23] WHO expert consultation (2004). Appropriate body-mass index for Asian populations and its implications for policy and intervention strategies. Lancet.

[24] Kluyver T, Ragan-Kelley B, Pérez F, Granger B, Bussonnier M, Frederic J, Kelley J (2016). Jupyter Notebooks – a publishing format for reproducible computational workflows. Positioning and Power in Academic Publishing: Players, Agents and Agendas.

[25] VanRossum G, Drake FL (2010). The Python Language Reference.

[26] R Core Team (2013). R: a language and environment for statistical computing. R Foundation for Statistical Computing.

[27] Waskom M, Botvinnik O, Hobson P, Halchenko Y, Lukauskas S, Cole JB, Warmenhoven J (2016). seaborn: v0.7.1 (June 2016). Zenodo.

[28] Marutho D, Handaka SH, Wijaya E, Muljono (2018). The determination of cluster number at k-mean using elbow method and purity evaluation on headline news.

[29] Ngeow J, Eng C (2021). Oh GxE! the complexity of body mass index and colon cancer risk. J Natl Cancer Inst.

[30] Campbell PT, Lin Y, Bien SA, Figueiredo JC, Harrison TA, Guinter MA, Berndt SI, Brenner H, Chan AT, Chang-Claude J, Gallinger SJ, Gapstur SM, Giles GG, Giovannucci E, Gruber SB, Gunter M, Hoffmeister M, Jacobs EJ, Jenkins MA, Le Marchand L, Li L, McLaughlin JR, Murphy N, Milne RL, Newcomb PA, Newton C, Ogino S, Potter JD, Rennert G, Rennert HS, Robinson J, Sakoda LC, Slattery ML, Song Y, White E, Woods MO, Casey G, Hsu L, Peters U (2021). Association of body mass index with colorectal cancer risk by genome-wide variants. J Natl Cancer Inst.

[31] Feliciano Elizabeth M Cespedes, Kroenke CH, Meyerhardt JA, Prado CM, Bradshaw PT, Kwan ML, Xiao J, Alexeeff S, Corley D, Weltzien E, Castillo AL, Caan BJ (2017). Association of systemic inflammation and sarcopenia with survival in nonmetastatic colorectal cancer: results from the C SCANS study. JAMA Oncol.

[32] Gilchrist SC, Howard VJ, Akinyemiju T, Judd SE, Cushman M, Hooker SP, Diaz KM (2020). Association of sedentary behavior with cancer mortality in middle-aged and older US adults. JAMA Oncol.

[33] Takemura N, Chan SL, Smith R, Cheung DST, Lin C (2021). The effects of physical activity on overall survival among advanced cancer patients: a systematic review and meta-analysis. BMC Cancer.

[34] Hardee JP, Porter RR, Sui X, Archer E, Lee I, Lavie CJ, Blair SN (2014). The effect of resistance exercise on all-cause mortality in cancer survivors. Mayo Clin Proc.

[35] Strasser B, Steindorf K, Wiskemann J, Ulrich CM (2013). Impact of resistance training in cancer survivors: a meta-analysis. Med Sci Sports Exerc.

[36] Singh B, Hayes SC, Spence RR, Steele ML, Millet GY, Gergele L (2020). Exercise and colorectal cancer: a systematic review and meta-analysis of exercise safety, feasibility and effectiveness. Int J Behav Nutr Phys Act.

[37] Kang DW, Fairey AS, Boulé Normand G, Field CJ, Wharton SA, Courneya KS (2021). Effects of exercise on cardiorespiratory fitness and biochemical progression in men with localized prostate cancer under active surveillance: the ERASE randomized clinical trial. JAMA Oncol.

