# Supplementary figures and images for: Investigation of the Trajectory of Muscle and Body Mass as a Prognostic Factor in Patients With Colorectal Cancer: Longitudinal Cohort Study
Source: JMIR Public Health Surveill. 2023 Mar 22;9:e43409. doi: 10.2196/43409 (PMC10131753; doi:10.2196/43409)

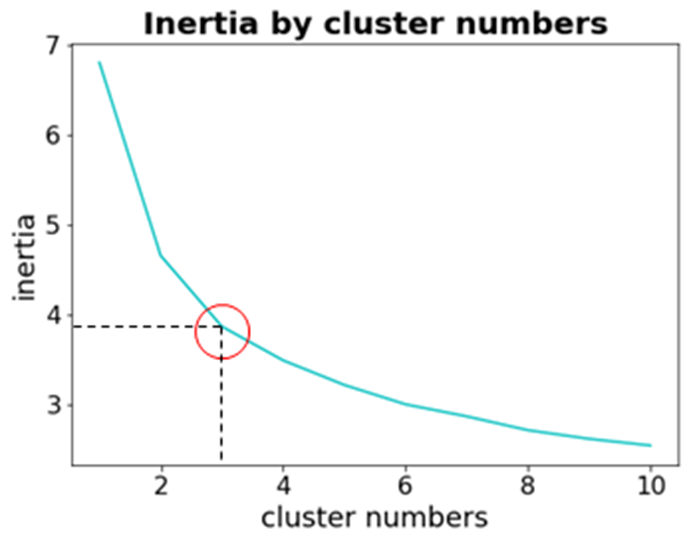

Supplement: Multimedia Appendix 1 [file publichealth_v9i1e43409_app1.png]

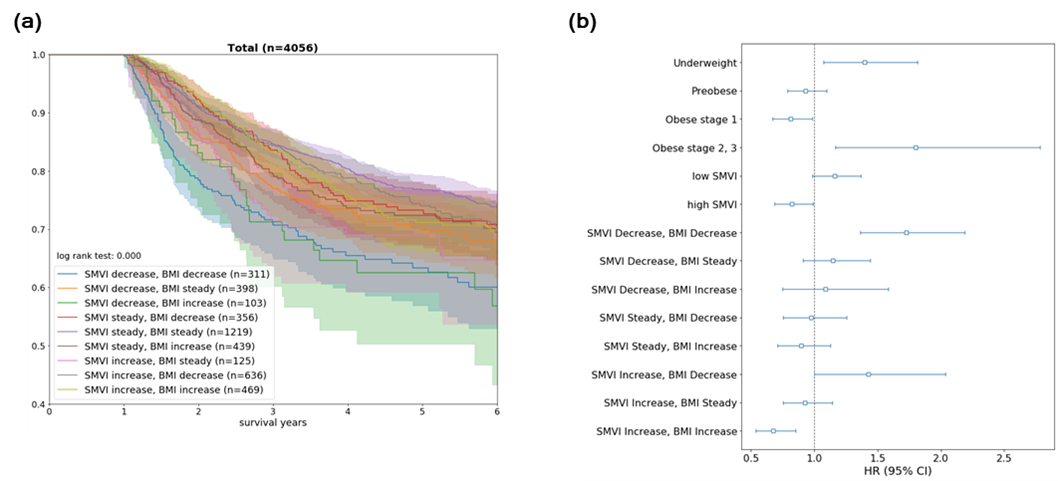

Supplement: Multimedia Appendix 7 [file publichealth_v9i1e43409_app7.png]
