# Supplementary material for: Investigation of the Trajectory of Muscle and Body Mass as a Prognostic Factor in Patients With Colorectal Cancer: Longitudinal Cohort Study
Source: JMIR Public Health Surveill. 2023 Mar 22;9:e43409. doi: 10.2196/43409 (PMC10131753; doi:10.2196/43409)
Supplement: Multimedia Appendix 2 [file publichealth_v9i1e43409_app2.docx]

**Multimedia Appendix 2.** Cox proportional hazard regression result within total patients. Adjusted variables were age at diagnosis (above or below 65 years); sex; stage; primary cancer location (colon or rectum); histology (adenocarcinoma or others); recurrence or metastasis; the administration of surgery, chemotherapy, or radiotherapy; baseline BMI (underweight, normal, preobese, obesity stage 1, or obesity stages 2-3); baseline SMVI (low, normal, or high); and patterns of BMI and SMVI (decreased, steady, or increased). SMVI: skeletal muscle volume index.

|  |  | **Hazard ratio** | **Lower 95% CI** | **Upper 95% CI** | ***P* value** |
| --- | --- | --- | --- | --- | --- |
| **Age at dx** | | | | | |
|  | <65 | 1 (Reference) | - | - | - |
|  | ≥65 | 1.30 | 1.14 | 1.49 | <.001 |
| **Sex** | | | | | |
|  | Male | 1 (Reference) | - | - | - |
|  | Female | 0.90 | 0.78 | 1.06 | .20 |
| **Stage (I, II, III, IV)** | | 1.29 | 1.15 | 1.45 | <.001 |
| **Primary location** | | | | | |
|  | Colon | 1 (Reference) | - | - | - |
|  | Rectum | 1.25 | 0.68 | 2.29 | .48 |
| **Histology** | | | | | |
|  | Adenocarcinoma | 1 (Reference) | - | - | - |
|  | Others | 1.44 | 0.99 | 2.10 | .06 |
| **Recur or metastasis** | | | | | |
|  | Yes | 11.71 | 9.07 | 15.12 | <.001 |
|  | No | 1 (Reference) | - | - | - |
| **Surgery** | | | | | |
|  | Yes | 0.23 | 0.20 | 0.27 | <.001 |
|  | No | 1 (Reference) | - | - | - |
| **CTx** | | | | | |
|  | Yes | 0.54 | 0.39 | 0.75 | <.001 |
|  | No | 1 (Reference) | - | - | - |
| **Rtx** | | | | | |
|  | Yes | 1.06 | 0.92 | 1.21 | .42 |
|  | No | 1 (Reference) | - | - | - |
| **Baseline BMI group** | | | | | |
|  | Underweight | 1.38 | 1.06 | 1.80 | .02 |
|  | Normal | 1 (Reference) | - | - | - |
|  | Preobese | 0.92 | 0.78 | 1.09 | .32 |
|  | Obese stage 1 | 0.80 | 0.66 | 0.97 | .02 |
|  | Obese stages 2-3 | 1.79 | 1.16 | 2.76 | .008 |
| **Baseline SMVI group** | | | | | |
|  | Low | 1.16 | 0.98 | 1.37 | .08 |
|  | Normal | 1 (Reference) | - | - | - |
|  | High | 0.82 | 0.68 | 0.99 | .04 |
| **BMI pattern** | | | | | |
|  | Decreased | 1.23 | 1.04 | 1.45 | .02 |
|  | Steday | 1 (Reference) | - | - | - |
|  | Increased | 0.83 | 0.71 | 0.97 | .02 |
| **SMVI pattern** | | | | | |
|  | Decreased | 1.31 | 1.11 | 1.54 | .001 |
|  | Steady | 1 (Reference) | - | - | - |
|  | Increased | 0.93 | 0.80 | 1.09 | .37 |
