# Supplementary material for: Investigation of the Trajectory of Muscle and Body Mass as a Prognostic Factor in Patients With Colorectal Cancer: Longitudinal Cohort Study
Source: JMIR Public Health Surveill. 2023 Mar 22;9:e43409. doi: 10.2196/43409 (PMC10131753; doi:10.2196/43409)
Supplement: Multimedia Appendix 3 [file publichealth_v9i1e43409_app3.docx]

**Multimedia Appendix 3.** Cox proportional hazard regression result within the decreased BMI group. Adjusted variables were age at diagnosis (above or below 65 years); sex; stage; primary cancer location (colon or rectum); histology (adenocarcinoma or others); recurrence or metastasis; the administration of surgery, chemotherapy, or radiotherapy; baseline BMI (underweight, normal, preobese, obesity stage 1, or obesity stages 2-3); baseline SMVI (low, normal, or high); and patterns of SMVI (decreased, steady, or increased). SMVI: skeletal muscle volume index.

|  |  | **Hazard ratio** | **Lower 95% CI** | **Upper 95% CI** | ***P* value** |
| --- | --- | --- | --- | --- | --- |
| **Age at dx** | |  |  |  |  |
|  | <65 | 1 (Reference) | - | - | - |
|  | ≥65 | 1.40 | 1.05 | 1.88 | .02 |
| **Sex** | |  |  |  |  |
|  | Male | 1 (Reference) | - | - | - |
|  | Female | 1.02 | 0.72 | 1.45 | .91 |
| **Stage (I, II, III, IV)** | | 1.30 | 1.01 | 1.66 | .04 |
| **Primary location** | |  |  |  |  |
|  | Colon | 1 (Reference) | - | - | - |
|  | Rectum | 1.70 | 0.75 | 3.88 | .20 |
| **Histology** | |  |  |  |  |
|  | Adenocarcinoma | 1 (Reference) | - | - | - |
|  | Others | 1.08 | 0.53 | 2.2 | .82 |
| **Recur or metastasis** | | |  |  |  |
|  | Yes | 9.84 | 5.83 | 16.63 | <.001 |
|  | No | 1 (Reference) | - | - | - |
| **Surgery** | |  |  |  |  |
|  | Yes | 0.25 | 0.17 | 0.37 | <.001 |
|  | No | 1 (Reference) | - | - | - |
| **CTx** | |  |  |  |  |
|  | Yes | 0.65 | 0.34 | 1.21 | .17 |
|  | No | 1 (Reference) | - | - | - |
| **Rtx** | |  |  |  |  |
|  | Yes | 0.81 | 0.59 | 1.10 | .17 |
|  | No | 1 (Reference) | - | - | - |
| **Baseline BMI group** | | |  |  |  |
|  | Underweight | 1.24 | 0.55 | 2.77 | .61 |
|  | Normal | 1 (Reference) | - | - | - |
|  | Preobese | 1.01 | 0.70 | 1.44 | .97 |
|  | Obese stage 1 | 0.82 | 0.57 | 1.19 | .30 |
|  | Obese stages 2-3 | 2.06 | 1.07 | 3.97 | .03 |
| **Baseline SMVI group** | | |  |  |  |
|  | Low | 1.11 | 0.77 | 1.61 | .58 |
|  | Normal | 1 (Reference) | - | - | - |
|  | High | 0.75 | 0.51 | 1.10 | .14 |
| **SMVI pattern** | |  |  |  |  |
|  | Decreased | 1.75 | 1.30 | 2.37 | <.001 |
|  | Steady | 1 (Reference) | - | - | - |
|  | Increased | 1.36 | 0.90 | 2.04 | 0.14 |
