# Supplementary material for: Investigation of the Trajectory of Muscle and Body Mass as a Prognostic Factor in Patients With Colorectal Cancer: Longitudinal Cohort Study
Source: JMIR Public Health Surveill. 2023 Mar 22;9:e43409. doi: 10.2196/43409 (PMC10131753; doi:10.2196/43409)
Supplement: Multimedia Appendix 4 [file publichealth_v9i1e43409_app4.docx]

**Multimedia Appendix 4.** Cox proportional hazard regression result within the steady BMI group. Adjusted variables were age at diagnosis (above or below 65 years); sex; stage; primary cancer location (colon or rectum); histology (adenocarcinoma or others); recurrence or metastasis; the administration of surgery, chemotherapy, or radiotherapy; baseline BMI (underweight, normal, preobese, obesity stage 1, or obesity stages 2-3); baseline SMVI (low, normal, or high); and patterns of SMVI (decreased, steady, or increased). SMVI: skeletal muscle volume index.

|  |  | **Hazard ratio** | **Lower 95% CI** | **Upper 95% CI** | ***P* value** |
| --- | --- | --- | --- | --- | --- |
| **Age at dx** | |  |  |  |  |
|  | <65 | 1 (Reference) | - | - | - |
|  | ≥65 | 1.35 | 1.12 | 1.63 | .002 |
| **Sex** | |  |  |  |  |
|  | Male | 1 (Reference) | - | - | - |
|  | Female | 0.83 | 0.67 | 1.03 | .10 |
| **Stage (I, II, III, IV)** | | 1.26 | 1.08 | 1.46 | .003 |
| **Primary location** | |  |  |  |  |
|  | Colon | 1 (Reference) | - | - | - |
|  | Rectum | 0.90 | 0.29 | 2.85 | .86 |
| **Histology** | |  |  |  |  |
|  | Adenocarcinoma | 1 (Reference) | - | - | - |
|  | Others | 1.38 | 0.82 | 2.34 | .23 |
| **Recur or metastasis** | | |  |  |  |
|  | Yes | 12.94 | 9.14 | 18.33 | <.001 |
|  | No | 1 (Reference) | - | - | - |
| **Surgery** | |  |  |  |  |
|  | Yes | 0.21 | 0.17 | 0.26 | <.001 |
|  | No | 1 (Reference) | - | - | - |
| **CTx** | |  |  |  |  |
|  | Yes | 0.56 | 0.36 | 0.86 | .009 |
|  | No | 1 (Reference) | - | - | - |
| **Rtx** | |  |  |  |  |
|  | Yes | 1.13 | 0.94 | 1.36 | .19 |
|  | No | 1 (Reference) | - | - | - |
| **Baseline BMI group** | | |  |  |  |
|  | Underweight | 1.85 | 1.29 | 2.67 | .001 |
|  | Normal | 1 (Reference) | - | - | - |
|  | Preobese | 0.92 | 0.73 | 1.15 | .46 |
|  | Obese stage 1 | 0.82 | 0.63 | 1.06 | .13 |
|  | Obese stages 2-3 | 1.43 | 0.67 | 3.09 | .36 |
| **Baseline SMVI group** | | |  |  |  |
|  | Low | 1.12 | 0.89 | 1.41 | .34 |
|  | Normal | 1 (Reference) | - | - | - |
|  | High | 0.86 | 0.66 | 1.11 | .25 |
| **SMVI pattern** | |  |  |  |  |
|  | Decreased | 1.12 | 0.89 | 1.42 | .33 |
|  | Steady | 1 (Reference) | - | - | - |
|  | Increased | 0.93 | 0.76 | 1.15 | .51 |
