# Supplementary material for: Investigation of the Trajectory of Muscle and Body Mass as a Prognostic Factor in Patients With Colorectal Cancer: Longitudinal Cohort Study
Source: JMIR Public Health Surveill. 2023 Mar 22;9:e43409. doi: 10.2196/43409 (PMC10131753; doi:10.2196/43409)
Supplement: Multimedia Appendix 5 [file publichealth_v9i1e43409_app5.docx]

**Multimedia Appendix 5.** Cox proportional hazard regression result within the increased BMI group. Adjusted variables were age at diagnosis (above or below 65 years); sex; stage; primary cancer location (colon or rectum); histology (adenocarcinoma or others); recurrence or metastasis; the administration of surgery, chemotherapy, or radiotherapy; baseline BMI (underweight, normal, preobese, obesity stage 1, or obesity stages 2-3); baseline SMVI (low, normal, or high); and patterns of SMVI (decreased, steady, or increased). SMVI: skeletal muscle volume index.

|  |  | **Hazard ratio** | **Lower 95% CI** | **Upper 95% CI** | ***P* value** |
| --- | --- | --- | --- | --- | --- |
| **Age at dx** | |  |  |  |  |
|  | <65 | 1 (Reference) | - | - | - |
|  | ≥65 | 1.11 | 0.85 | 1.45 | .46 |
| **Sex** | |  |  |  |  |
|  | Male | 1 (Reference) | - | - | - |
|  | Female | 0.93 | 0.69 | 1.25 | .62 |
| **Stage (I, II, III, IV)** | | 1.36 | 1.07 | 1.74 | .01 |
| **Primary location** | |  |  |  |  |
|  | Colon | 1 (Reference) | - | - | - |
|  | Rectum | 0.67 | 0.09 | 4.87 | .70 |
| **Histology** | |  |  |  |  |
|  | Adenocarcinoma | 1 (Reference) | - | - | - |
|  | Others | 2.48 | 1.00 | 6.15 | .05 |
| **Recur or metastasis** | | |  |  |  |
|  | Yes | 10.78 | 6.19 | 18.77 | <.001 |
|  | No | 1 (Reference) | - | - | - |
| **Surgery** | |  |  |  |  |
|  | Yes | 0.26 | 0.19 | 0.35 | <.001 |
|  | No | 1 (Reference) | - | - | - |
| **CTx** | |  |  |  |  |
|  | Yes | 0.32 | 0.13 | 0.76 | .01 |
|  | No | 1 (Reference) | - | - | - |
| **Rtx** | |  |  |  |  |
|  | Yes | 1.19 | 0.91 | 1.56 | .21 |
|  | No | 1 (Reference) | - | - | - |
| **Baseline BMI group** | | |  |  |  |
|  | Underweight | 1.12 | 0.72 | 1.74 | .62 |
|  | Normal | 1 (Reference) | - | - | - |
|  | Preobese | 0.89 | 0.62 | 1.27 | .52 |
|  | Obese stage 1 | 0.81 | 0.53 | 1.25 | .34 |
|  | Obese stages 2-3 | 1.60 | 0.58 | 4.41 | .37 |
| **Baseline SMVI group** | | |  |  |  |
|  | Low | 1.30 | 0.93 | 1.81 | .12 |
|  | Normal | 1 (Reference) | - | - | - |
|  | High | 0.80 | 0.55 | 1.17 | .26 |
| **SMVI pattern** | |  |  |  |  |
|  | Decreased | 1.32 | 0.88 | 1.98 | .18 |
|  | Steady | 1 (Reference) | - | - | - |
|  | Increased | 0.73 | 0.55 | 0.97 | .03 |
