# Supplementary material for: Investigation of the Trajectory of Muscle and Body Mass as a Prognostic Factor in Patients With Colorectal Cancer: Longitudinal Cohort Study
Source: JMIR Public Health Surveill. 2023 Mar 22;9:e43409. doi: 10.2196/43409 (PMC10131753; doi:10.2196/43409)
Supplement: Multimedia Appendix 6 [file publichealth_v9i1e43409_app6.docx]

**Multimedia Appendix 6.** Cox proportional hazard regression result for hazard ratio heat map. Adjusted variables were age at diagnosis (above or below 65 years); sex; stage; primary cancer location (colon or rectum); histology (adenocarcinoma or others); recurrence or metastasis; the administration of surgery, chemotherapy, or radiotherapy; baseline BMI (underweight, normal, preobese, obesity stage 1, or obesity stages 2-3); baseline SMVI (low, normal, or high); and 9 patient groups divided by three 1-year BMI trajectories (decreased, steady, or increased) and three 1-year SMVI trajectories (decreased, steady, or increased). SMVI: skeletal muscle volume index.

|  |  | **Hazard ratio** | **Lower 95% CI** | **Upper 95% CI** | ***P* value** |
| --- | --- | --- | --- | --- | --- |
| **Age at dx** | |  |  |  |  |
|  | <65 | 1 (Reference) | - | - | - |
|  | ≥65 | 1.30 | 1.14 | 1.49 | <.001 |
| **Sex** | |  |  |  |  |
|  | Male | 1 (Reference) | - | - | - |
|  | Female | 0.91 | 0.78 | 1.06 | .21 |
| **Stage (I, II, III, IV)** | | 1.29 | 1.15 | 1.45 | <.001 |
| **Primary location** | |  |  |  |  |
|  | Colon | 1 (Reference) | - | - | - |
|  | Rectum | 1.20 | 0.65 | 2.20 | .57 |
| **Histology** | |  |  |  |  |
|  | Adenocarcinoma | 1 (Reference) | - | - | - |
|  | Others | 1.45 | 1.00 | 2.12 | .05 |
| **Recur or metastasis** | | |  |  |  |
|  | Yes | 11.86 | 9.18 | 15.32 | <.001 |
|  | No | 1 (Reference) | - | - | - |
| **Surgery** | |  |  |  |  |
|  | Yes | 0.23 | 0.19 | 0.27 | <.001 |
|  | No | 1 (Reference) | - | - | - |
| **CTx** | |  |  |  |  |
|  | Yes | 0.53 | 0.38 | 0.74 | <.001 |
|  | No | 1 (Reference) | - | - | - |
| **Rtx** | |  |  |  |  |
|  | Yes | 1.06 | 0.93 | 1.21 | .52 |
|  | No | 1 (Reference) | - | - | - |
| **Baseline BMI group** | | |  |  |  |
|  | Underweight | 1.40 | 1.07 | 1.82 | .01 |
|  | Normal | 1 (Reference) | - | - | - |
|  | Preobese | 0.93 | 0.79 | 1.10 | .39 |
|  | Obese stage 1 | 0.81 | 0.67 | 0.98 | .03 |
|  | Obese stages 2-3 | 1.80 | 1.17 | 2.78 | .008 |
| **Baseline SMVI group** | | |  |  |  |
|  | Low | 1.16 | 0.98 | 1.37 | .08 |
|  | Normal | 1 (Reference) | - | - | - |
|  | High | 0.82 | 0.68 | 0.99 | .04 |
| **Pattern group** | |  |  |  |  |
|  | SMVI Decreased, BMI Decreased | 1.73 | 1.36 | 2.19 | <.001 |
|  | SMVI Decreased, BMI Steady | 1.15 | 0.91 | 1.44 | .24 |
|  | SMVI Decreased, BMI Increased | 1.09 | 0.75 | 1.58 | .65 |
|  | SMVI Steady, BMI Decreased | 0.97 | 0.75 | 1.26 | .83 |
|  | SMVI Steady, BMI Steady | 1 (Reference) | - | - | - |
|  | SMVI Steady, BMI Increased | 0.90 | 0.71 | 1.13 | .35 |
|  | SMVI Increased, BMI Decreased | 1.43 | 1.00 | 2.04 | .049 |
|  | SMVI Increased, BMI Steady | 0.93 | 0.75 | 1.14 | .48 |
|  | SMVI Increased, BMI Increased | 0.68 | 0.54 | 0.85 | .001 |
